# Integrating Pharmacological and Computational Approaches for the Phytochemical Analysis of *Syzygium cumini* and Its Anti-Diabetic Potential

**DOI:** 10.3390/molecules27175734

**Published:** 2022-09-05

**Authors:** Fatima Rashid, Anam Javaid, Mahmood-ur-Rahman, Usman Ali Ashfaq, Muhammad Sufyan, Abdulrahman Alshammari, Metab Alharbi, Muhammad Atif Nisar, Mohsin Khurshid

**Affiliations:** 1 Department of Bioinformatics and Biotechnology, Government College University, Faisalabad 38000, Pakistan; 2 Department of Pharmacology and Toxicology, College of Pharmacy, King Saud University, P.O. Box 2455, Riyadh 11451, Saudi Arabia; 3 College of Science and Engineering, Flinders University, Bedford Park 5042, Australia; 4 Department of Microbiology, Government College University, Faisalabad 38000, Pakistan

**Keywords:** diabetes, molecular docking, phytochemicals

## Abstract

Diabetes mellitus (DM) is a metabolic disease caused by improper insulin secretion leading to hyperglycemia. *Syzygium cumini* has excellent therapeutic properties due to its high levels of phytochemicals. The current research aimed to evaluate the anti-diabetic potential of *S. cumini* plant’s seeds and the top two phytochemicals (kaempferol and gallic acid) were selected for further analysis. These phytochemicals were selected via computational tools and evaluated for α-Glucosidase inhibitory activity via enzymatic assay. Gallic acid (IC_50_ 0.37 µM) and kaempferol (IC_50_ 0.87 µM) have shown a stronger α-glucosidase inhibitory capacity than acarbose (5.26 µM). In addition, these phytochemicals demonstrated the highest binding energy, hydrogen bonding, protein–ligand interaction and the best MD simulation results at 100 ns compared to acarbose. Furthermore, the ADMET properties of gallic acid and kaempferol also fulfilled the safety criteria. Thus, it was concluded that *S. cumini* could potentially be used to treat DM. The potential bioactive molecules identified in this study (kaempferol and gallic acid) may be used as lead drugs against diabetes.

## 1. Introduction

DM is a group of diseases known as diabetes, which can be combined with hypertension, heart diseases and abnormal level of triglycerides and cholesterol. Hyperglycemia is a symptom of diabetes, which is induced by insufficient, or lack of, insulin secretion [1,2]. In diabetic patients, chronic hyperglycemia may be associated with the failure, damage and dysfunction of various body organs [3,4]. It primarily affects the kidneys (nephropathy), heart via cardiovascular diseases, blood vessels, eyes (retinopathy) and nerves (neuropathy) [5,6]. International Diabetes Federation (IDF) statistics indicate that 1 in every 11 has diabetes globally. The prevalence of DM is from 7.66% to 11%. It has been projected that this will increase to 15% in 2030. Diabetes is a progressive disease that gradually wears out body functions and vital body parts. According to the WHO, there are about 422 million diabetes patients worldwide [7]. 

Many oral anti-hyperglycemic drugs (such as sulfonylureas, glucosidase inhibitors, etc.) have been identified to improve glycemic control [8]. Inhibitors of the α-glucosidase enzyme, for instance, miglitol, acarbose and many more, inhibit an intestinal enzyme’s role (α-glucosidase), which acts in a dose-dependent manner to convert complex polysaccharides into simpler sugars [9]. While α-glucosidase controlling agents can effectively manage the level of postprandial hyperglycemia, they also have many unwanted side reactions, including inflating, flatulence, nausea and gastroenteritis [10]. 

Various phytochemicals such as phenolic compounds, luteolin, flavonoids and alkaloids make medicinal plants extremely beneficial [11]. Many heterocyclic compounds, including ferulic acid, kaempferol, ellagic acid, caffeic acid, gallic acid, synergic acid and others, are attracting attention for their anti-diabetic, anti-inflammatory, antifungal, anticancer and antibacterial properties [12,13,14]. Previous reports have shown that gallic acid and kaempferol are suitable α-glucosidase inhibitors [15,16]. These phytochemicals are found in various fruits, vegetables and lentils [17]. Gallic acid and kaempferol are present in plants as free acids, methylated forms and catechin conjugated derivatives. 

Gallic acid is a derivative of polyphenolic phytocompound, named 3,4,5-trihydroxy benzoic acid and is frequently present in various fruits such as grapes and strawberries [18]. Several reports showed that gallic acid has significant biological and pharmacological activities [19] and is also a suitable diabetes inhibitor [18,20]. Kaempferol is a flavonoid derivative in many vegetables and fruits [21]. This phytochemical also has excellent health significance due to its anti-hyperglycemic and anticancer effects [22,23]. 

There is still a great challenge in treating diabetes without any side effects. Medicinal plants are a great source for the discovery of new anti-diabetic drugs with pharmaceutical importance [24]. Metformin is frequently prescribed to treat diabetes and control hyperglycemic condition and is prepared from galegine [25]. This anti-diabetic agent is a plant-derived compound isolated from *Galega officinalis*. Deoxy-nojirimycin (DNJ) is also a plant derived anti-diabetic compound isolated from *Morus alba* L. extract [26]. Recent studies have identified many phytochemicals from *S.cumini* with potential inhibitory activity against α-glucosidases and amylases [27]. 

*Syzygium cumini* is an evergreen tropical plant that belongs to the *Myrtaceae* family, found in the Indian subcontinent and Southeast Asia, Queensland, Pakistan, Bangladesh and China. Its common name is Jamun, Java Plum and Black Plum [28]. According to current knowledge, the healing or health-promoting properties of *S. cumini* seed are due to the bioactivity of a variety of phytochemicals, including oleic acid, hexadecenoic acid, corilagin and eicosane [29]. Several activities, including anti-microbial, hypolipidemic, anti-inflammatory, antiulcerogenic, gastroprotective, immunomodulatory, antioxidant and many others, are associated. *Syzigium cumini* seeds have been traditionally used for health purposes in Ayurveda, Unani and Chinese medicines. The seed powder of *S. cumini* has increasingly been used for diabetes management by Bangladeshis during recent years [28]. 

The foremost objective of the current research is to explore the anti-hyperglycemic activity of *S. cumini* seed and its active phenolic ingredients. The potent phytochemicals such as kaempferol and gallic acid were analyzed in silico, including MD simulation and ADMET. The present work aims to study the enzyme kinetics and protein-ligand relationships of kaempferol and gallic acid with α-glucosidase inhibition. Enzymatic assays were also performed to demonstrate that these phytochemicals have considerably higher inhibitory activity than standard acarbose.

## 2. Results

### 2.1. Enzyme Inhibition Assay

**Figure 1:**
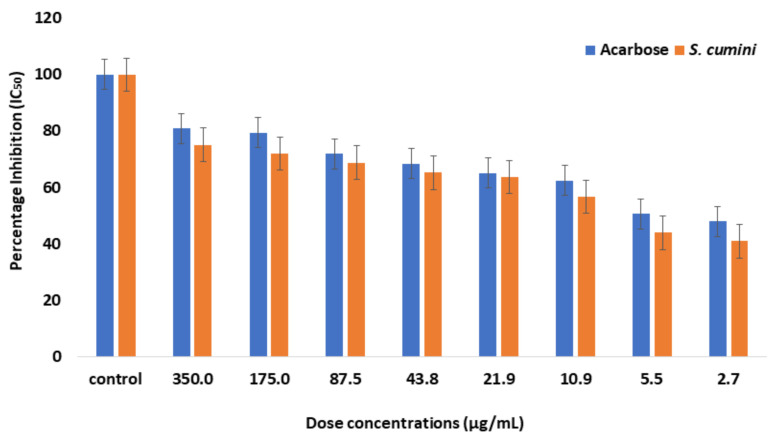
The percentage inhibition of α-glucosidase by increasing concentrations of acarbose and *S. cumini* extract. The resulting IC_50_ is 8.07 (µg/mL) for *S. cumini*. This data was taken in triplicates (n = 3) with SD ± mean values.

When investigating the inhibitory activity of *S. cumini* against α-glucosidase, PNPG was used as the substrate. *S. cumini* exhibited IC_50_ at a concentration of 8.07 µg/mL, while acarbose exhibited IC_50_ at a concentration of 5.26 µM (Table 1) (Figure 1). 

### 2.2. Molecular Docking

**Figure 2:**
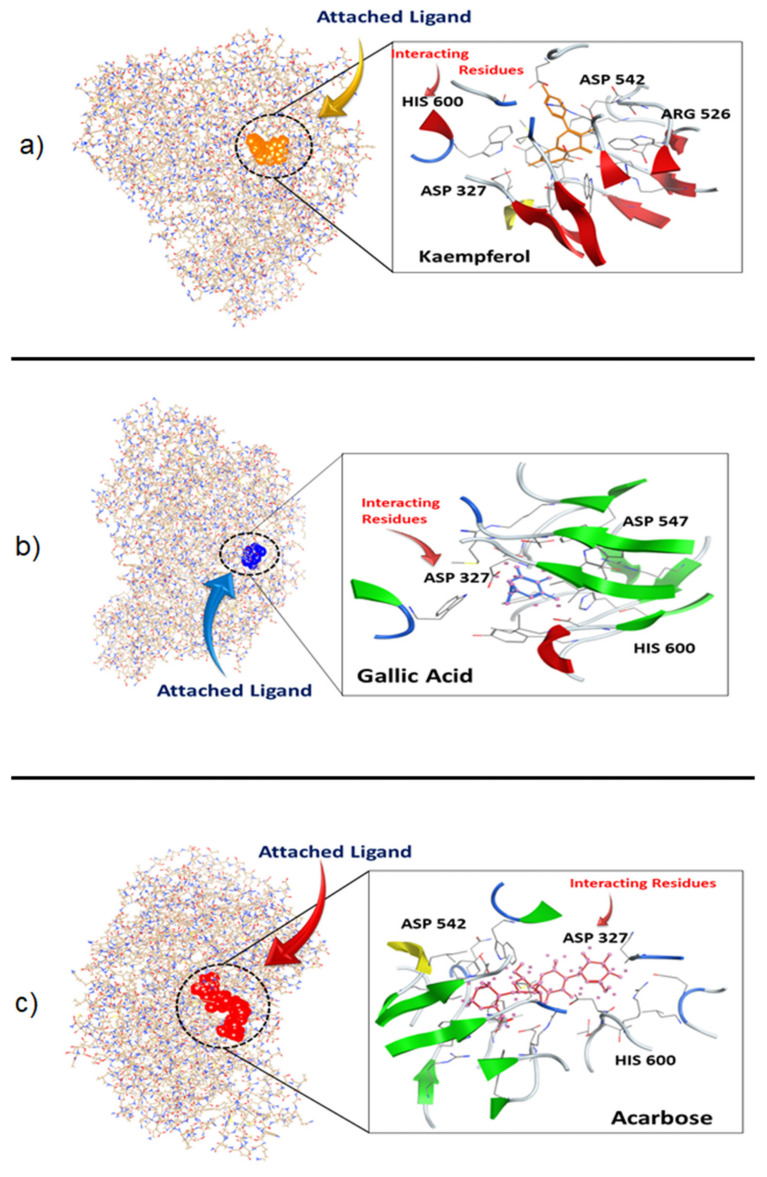
2D and 3D illustration of selected docked complex: Kaempferol (**a**), Gallic Acid (**b**) and Acarbose (**c**) against Maltase-glucoamylase, an α-glucosidase enzyme.

The three-dimensional structure of α-glucosidase was retrieved from PDB (PDB ID: 2QMJ). This structure was optimized via energy minimization, via removal of ligand and hydrogen atoms that could interfere with the interaction and do not permit the function of α-glucosidase. Docking was done with a ready to dock library of about 81 phytochemicals. The site finder tool of MOE was used to identify all of the residues involved in the reaction of the α-glucosidase with other ligands or proteins. When the phytochemical library was docked against PDB: 2QMJ, the compounds were ranked based on the highest bond pocket occupancy with the lowest Gibbs free energy, the strength of hydrogen bonding and the potential non-covalent interactions. Of the 81 phytochemical products, the top two phytochemicals were selected based on lower RMSD values, maximum ligand binding sites and minimum S-score. Kaempferol and gallic acid showed favorable binding energy ranging from −11.2 Kcal/mol to −10.3 Kcal/mol compared to reference drug acarbose with −12.6 Kcal/mol (Table 2). As a result of its maximum binding score of −11.2 Kcal/mol, kaempferol was the most effective compound for all selected binding. It was successfully docked with the Asp547, Asp327 and His600 residues involved, which are also present at the selected active site (Figure 2). 

### 2.3. Drug Scan/ADMET Results

In the Molinspiration server (accessed on 24 May 2022), an ADMET-based scan tool is used to determine whether the proposed α-glucosidase inhibitors are drug-like. Except for acarbose, all the shortlisted candidates have zero violations of Ro5 and standard ADMET characteristics, such as molecular weight (MW), that are acceptable for pharmaceutical use (Table 3). As shown in Table 3, gallic acid and kaempferol fulfilled Lipinski’s rules (RO5). According to this rule, MW should be less than 500 g/mol, MlogP less than 5, number of hydrogen bond acceptors less than 10, and hydrogen bond donors less than 5. Gallic acid and kaempferol showed positive results as compared to acarbose. The Swiss ADME server (http://www.swissadme.ch/index.php) (accessed on 24 May 2022), used to evaluate further the drug likeness of the compound, was used to analyze all the candidate compounds for their ADME characteristics. (Table 4). ADMET profiling is very important for safe drug delivery. Gallic acid and kaempferol showed high solubility in GI track compared to acarbose with poor brain-blood barrier (BBB) solubility. These phytochemicals cannot cause any adverse effect on the central nervous system. 

**Figure 3:**
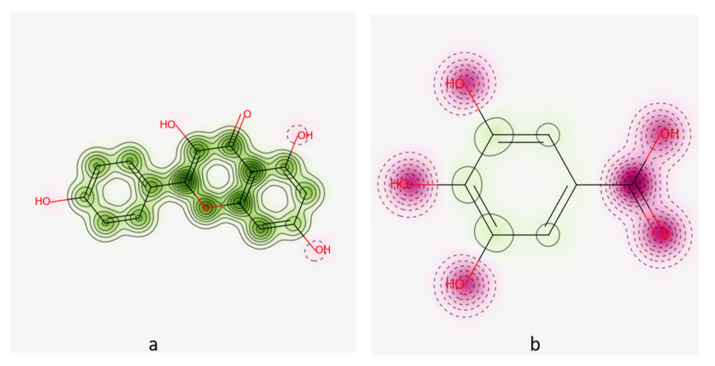
Cardiotoxicity analysis via Pred-hERG software (**a**) Kaempferol (**b**) Gallic acid.

Cardiotoxicity was also analyzed and predicted that kaempferol and gallic acid were non-cardiotoxic, with 60% confidence interval and no applicable domain (AD) (value 0.19 and limit 0.26). GUSAR software was used to predict the toxicity clearance, classification of toxicity and oral dose (LD50). Gallic acid and kaempferol showed applicable domain. Both drugs were safe for the body (Figure 3).

### 2.4. MD Simulation

Phytochemicals of significant importance, such as kaempferol and gallic acid, were reanalyzed by MD simulation using Desmond software. During 100 ns molecular dynamics simulation, time dependent changes were examined. The structural and dynamic alterations were monitored by observing the root mean square (RMSF) and root mean square fluctuation (RMSD). The phytochemicals kaempferol and gallic acid derived from *S. cumini* presented stable complexes with α-glucosidase (PDB ID: 2QMJ).

#### 2.4.1. Root Mean Square Deviation and Root Mean Square Fluctuation 

For analysis of the conformational dynamics of protein-ligand complexes up to 100 ns, MD simulation was performed to calculate the RMSD and RMSF values of kaempferol, gallic acid and acrobose as backbone atoms of α-glucosidase. Root mean square deviation is the average distance between the atoms of aligned protein and ligand during molecular docking and simulation. Root mean square fluctuation (RMSF) is a statistical measurement similar to the root mean square deviation (RMSD). Before calculating the RMSD and RMSF, ligand and protein were aligned. RMSD and RMSF was calculated by an automated process using the Desmond.

**Figure 4:**
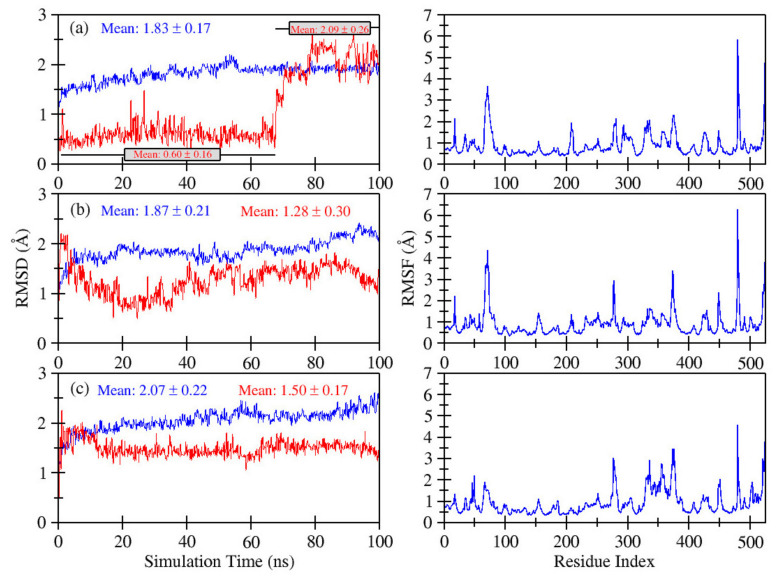
MD simulation interaction diagrams of 100 ns trajectory showing RMSD plot of kaempferol/α-glucosidase (**a**), gallic acid/α-glucosidase complex (**b**) and acarbose/αa-glucosidase (**c**). RMSF trajectory plot of kaempferol/α-glucosidase (**a**), gallic acid/α-glucosidase complex (**b**) and acarbose/α-glucosidase (**c**), respectively.

RMSD plots for kaempferol/α glucosidase, gallic acid/α glucosidase and acarbose/α-glucosidase are shown in Figure 4. All three simulations run on 100 ns. During the simulation, the RMSD plot for kaempferol/α glucosidase (a; blue line for protein) showed a slight increase until 55 ns and then stayed stable till the end. The mean RMSD value was observed as 1.83 ± 0.17 Å while kaempferol (a; red line for ligand) showed a minor deviation of 0.5 Å RMSD with mean value 0.60 ± 0.16 sudden increase after 68 ns, rising to 2.2 Å, after reaching 80 ns. The mean RMSD value was observed for ligand as 2.09 ± 0.26 Å after 68 ns. On the other hand, the RMSD plot for gallic acid/α glucosidase (b; blue line) showed a slight increase for the first 5 ns and was then stable till 84 ns with few fluctuations and again shows a consistent increase at the end of the simulation with a mean RMSD value of 1.87 ± 0.21 Å. While observing the ligand (gallic acid) (b; red line), it showed a sudden decrease till 38 ns and then stayed stable, with a small decrease at the end of simulation on 100 ns and with a mean RMSD 1.28 ± 0.30 Å. Figure c’s acarbose/α-glucosidase graph shows that both ligand and protein had no change and remained stable till the first 10 ns; later, protein α glucosidase (c; blue line) showed a consistently small increase until the end of the simulation with mean RMSD of 2.07 ± 0.22 Å and the ligand acarbose (c; red line) stayed stable with a sudden small decrease at 10 ns and mean RMSD of 1.50 ± 0.17 Å.

Overall, all complexes exhibited small deviation values (1.28 ≥ RMSD (av) ≤ 1.87 Å) from the initial structure, except two (c; blue line and a; red line), where the RMSD value was slightly higher. 

In addition, the metrics of RMSF were used to calculate the fluctuation residues along the protein chain. The RMSF peaks calculate the area of protein where residues fluctuate at a maximum over the simulation trajectory. RMSD shows the average positional distance between the entire system but RMSF calculates the individual residue flexibility, or is useful to calculate how much a particular residue moves during the simulation time. The RMSF peaks of the docked kaempferol/α-glucosidase and gallic acid/α-glucosidase complexes are shown in Figure 4. The RMSF peaks of kaempferol/α-glucosidase and gallic acid/α-glucosidase complexes were noticed between the ranges of 0.4 ± 2.3 Å, 0.8 ± 2.0 Å. The RMSF values of both complexes showed small deviations. RMSF values showed that protein remained stable during ligand binding with protein.

#### 2.4.2. Radius of Gyration (Rg) and Solvent Accessible Surface Area (SASA) 

**Figure 5:**
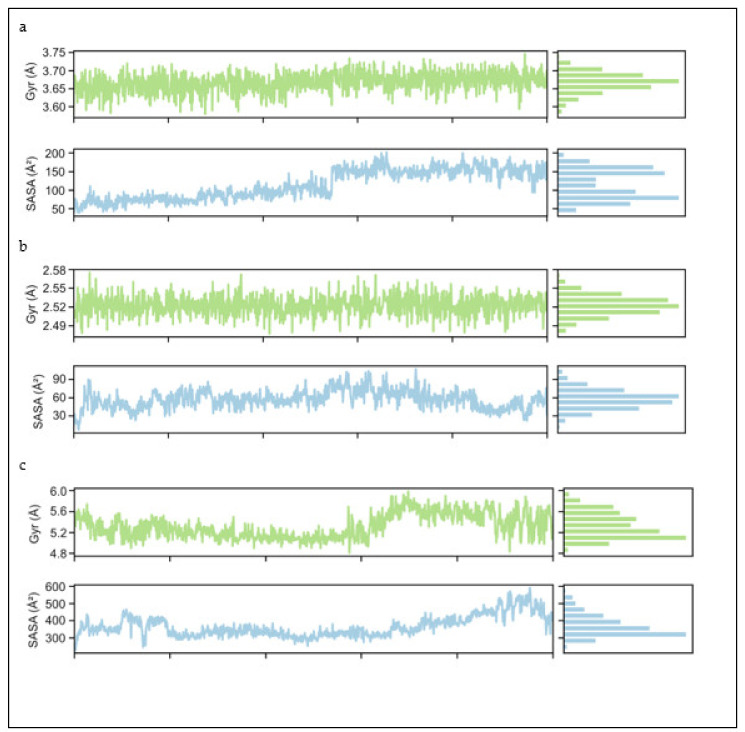
The time frame evolution against the radius of gyration (rGyr) for kaempferol with alpha-glucosidase (**a**), gallic acid with alpha-glucosidase (**b**) acarbose with alpha-glucosidase (**c**) and solvent accessible surface area (SASA) kaempferol, with alpha-glucosidase (**a**), gallic acid with alpha-glucosidase (**b**) and acarbose with alpha-glucosidase (**c**), respectively.

MD simulation was performed on three complexes: kaempferol/α-glucosidase (a), gallic acid/α-glucosidase complex (b) and Acarbose/αa-glucosidase (c) All the graphs in Figure 5 present the properties of all three ligands concerning their radius of gyration and Solvent Accessible Surface Area (SASA). Hence, these parameters during MD simulation evaluated the compactness of the receptor-docked complexes by the calculation of radius of gyration (Rg). The results showed that the Rg values of kaempferol stayed stable until the end of the simulation, with only a minor increase from start to end. Therefore, by looking at the overall progress, the observed mean value of Kaempferol was 3.67 ± 0.05. Examining the Solvent Accessible Surface Area (SASA) plot shows that it starts from the value of 60 Ᾰ and with a small increase reaches 100 Å by half of the simulation, then suddenly increases to 150 Å and stays stable until the end. The gallic acid (b) graph shows that Rg started from 2.53 ± 0.03 and remained stable until the end of simulation. On the other hand, while observing the SASA plot, it decreased from the start until 5 ns, suddenly increases up to 70 and then stays stable until the end with many fluctuations. Looking at the third ligand, acarbose, a sudden decrease from the start then remains stable till 60 ns and then starts increasing, becoming stable from 5.8 Ᾰ until the end with a lot of fluctuation in the last 20 ns. The mean values 5.4 ± 0.04 revealed the full compactness of acarbose with protein. In the SASA plot for acarbose, there is much variability in the first 20 ns, while it is very stable from 20 to 60 ns and then starts increasing as it moves forward until a sudden decrease just at the end of graph. The analysis confirmed the compactness of the native protein docked complexes with Rg average. Comparative Rg results revealed folding behaviour of compounds. Therefore, looking at the overall average mean value for all three ligands, i.e., 3.86 ± 0.04, shows the high compactness of these three ligands with α-glucosidase.

#### 2.4.3. Protein–Ligand Interaction 

**Figure 6:**
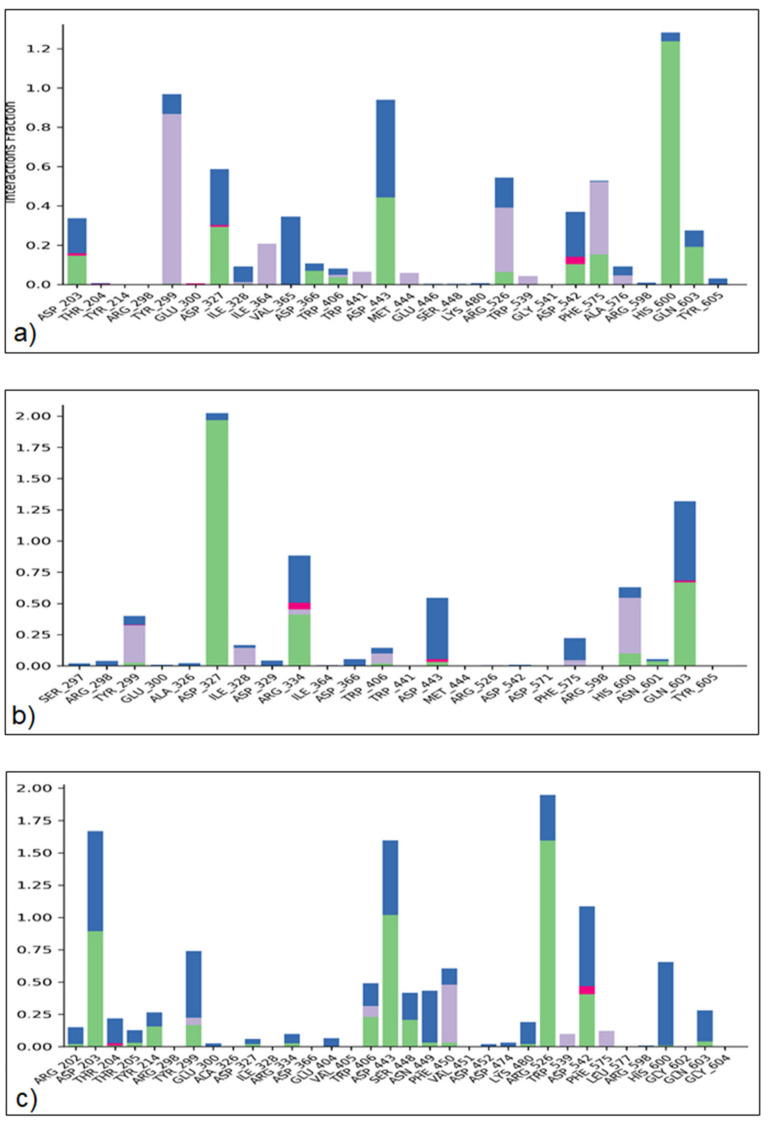
Protein-ligand contact interaction profile analyzed for kaempferol/alpha-glucosidase (**a**), gallic acid/alpha-glucosidase complex (**b**) and acarbose/alpha-glucosidase (**c**), respectively. Green = hydrogen bonding, pink = ionic interaction, grey = hydrophobic interaction and blue = water bridges.

The ability to predict the binding pocket of docked compounds to the target protein’s binding site requires atomic-level information. The different intermolecular interactions, such as H-bonds, H_2_O bridges, hydrophobic and ionic interactions, were examined over 100 ns of the MD simulation analysis for critical mode evaluation (Figure 6). The data showed that kaempferol made strong H-bonding with amino acids ASP-203, ASP-327, ASP-366, TRP-406, ASP-443, ARG-526, ASP-542, PHE-575, HIS-600 and GLN-603. This potent phytochemical also showed strong hydrophobic interactions with TYR-299, ILE-328, ILE-364, TRP-441, MET-444, ARG-526, TRP-539, PHE-575, ALA-576 and ionic strength with ASP-203, ASP-327 and ASP-542. The other potent compound, gallic acid, also showed maximum H-bonding with TRY-299, ASP327, ARG-334, TRP-406, ASP-443, HIS-600, ASN-601, GLN-603 amino acid residues. Gallic acid showed strong hydrophobic interactions with TRY-299, ILE-328, ARG-334, TRP-406, PHE-575, HIS-600 and ionic strength ARG-334, ASP-443 and GLN-603 residues (Figure 6).

### 2.5. Enzyme Inhibition Assay for Bioactive Phytochemicals

**Figure 7:**
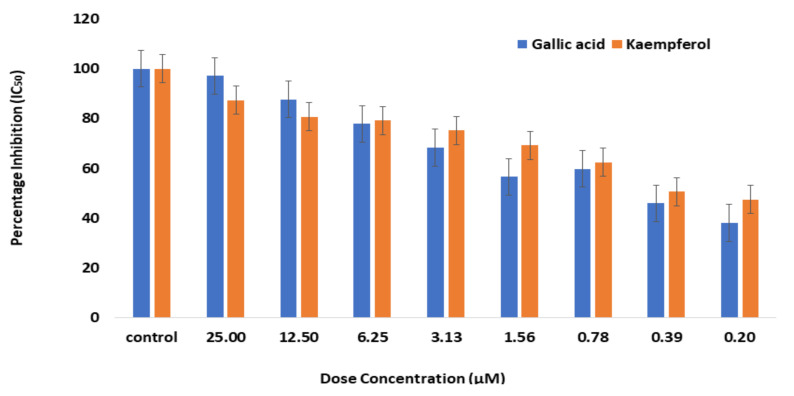
The percentage inhibition of α-glucosidase by increasing concentrations of gallic acid and kaempferol. The resulting IC_50_ is 0.37 µM for gallic acid and 0.87 µM for kaempferol. This data was taken in triplicates (n = 3) with SD ± mean values.

After demonstrating a potent in silico interaction, kaempferol and gallic acid were evaluated for inhibitory activity against α-glucosidase. The inhibitory activities of the kaempferol and gallic acid against α-glucosidase were compared with *S. cumini* extract. The substrate used in the experiment was p-Nitrophenyl-α-D-glucopyranoside (PNPG). Glucosidase could hydrolyze PNPG to PNP, which has a maximum absorbance at 405 nm. The inhibitory activity of α-glucosidase in the bioactive phytochemicals can be assessed based on PNP production. *S. cumini* showed IC_50_ at a concentration of 8.07 µg/mL, but gallic acid and kaempferol showed IC_50_ at a concentration of 0.37 µM and 0.87 µM, respectively (Figure 7). However, the gallic acid showed strong α-glucosidase at lower concentration (IC_50_ 0.37 µM) than kaempferol (IC_50_ 0.87 µM). 

## 3. Discussion

*Syzygium cumini,* commonly known as java plum, belongs to the family *Myrtaceae* [29]. Approximately 1200–1800 species are found in this family [30]. *S. cumini* was appreciated for its therapeutic ability in Siddha, Ayurveda and Unani medications. The entire plant is used in various conventional medical systems in India. *S. cumini* seeds are used in Unani as an astringent, diuretic, to avoid urinary discharge and as a diabetes remedy [29,31]. Various studies have shown that *S. cumini*’s seeds are a rich source of flavonoids, polyphenolic compounds, such as ferulic acid, ascorbic acid, gallic acid, ellagic acid, synergic acid, caffeic acid, etc. and have anti-diabetic properties due to the inhibition of intestinal α-glucosidases [32]. Among these phytochemicals, gallic acid and kaempferol are well known to boost glucose translocation via GLUT4 and glucose uptake properties [33].

Enzymes which catalyze the absorption of digested dietary carbohydrates from the small intestine, especially their glucose component, are called glucosidases [34]. When investigating the inhibitory effect of *S. cumini* against α-glucosidase, the PNPG substrate was used. In *S. cumini*, the inhibitory concentration (IC_50_) was 8.07 µg/mL and the IC_50_ of acarbose was 5.26 µM. *Senna surattensis* [34], *Bergenia ciliate, Cornus capitate, Haw* [35] and many more plants showed better anti-diabetic results than commercially available anti diabetic drugs. 

Drug designing has been revolutionized by in silico analysis and the complications and total costs of the conventional drug design process have been efficiently reduced. New therapies and applications are being found and circulated in enormous numbers due to the widespread use of more powerful databases, software and tools in bioinformatics. [36,37,38].

These phytocompounds were retrieved from pubchem IDs and docked with α-glucosidase to discover their attraction as inhibitors. Based on the minimum energy and best docking score, those with the top conformations were selected from 81 molecules. Our results demonstrated gallic acid and kaempferol’s potential and considerable interactions with α-glucosidase residues.

Current research shows that two bioactive phytochemicals have strong possible interactions and have important hydrophobic contact with α-glucosidase. Based on “Ro5,” these two potential compounds’ drug-likeliness and molecular properties were evaluated [39]. When high-performance and rapid ADMET profiling analyses are developed, they can aid in identifying active lead compounds during the early stages of the drug discovery process [40]. The ADMET compound profiling showed that the absorption of all compounds has no adverse effect. The characteristics of ADMET-related potential compounds have been demonstrated in several models, including P-glycoprotein substrates, BBB penetration and gastrointestinal uptake. Gallic acid and kaempferol showed high GI absorbance with no BBB penetrations compared to acarbose and these phytochemicals may not be able to create any harmful or adverse side effects. Similarly, gallic acid and acarbose are not susceptible for P-gp substrate but kaempferol showed positive results for P-gp substrate. P-glycoproteins have been used to transport the drug into targeted organs [41]. CYP enzymes are important for drug–drug interaction, and both kaempferol and gallic acid showed inhibition against CYP3A4 inhibitor. These results indicated that gallic acid and kaempferol can make drug–drug interactions with CYP3A4. These phytochemicals did not show any toxicity, such as cardiotoxicity, and were examined as non-inhibitor of hERG [42]. 

According to the results of this study, we selected the top two most potent phytochemicals based on their lowest binding energies, lowest RMSD value and highest binding affinity. Docking kaempferol and gallic acid results showed that these compounds have binding energy −11.2 Kcal/mol to −10.3 Kcal/mol, respectively, with many H-bonds. Asp542, Arg526, Asp327 and His600 residues of glucosidase bind with kaempferol and gallic acid. Several studies have also revealed that kaempferol and gallic acid have strong inhibitory properties and strong binding with α-glucosidase [43]. MD simulation was also performed to check the stability of both phyto-complexes: α-glucosidase and gallic acid: α-glucosidase at 100 ns along with α-glucosidase, which showed stable complexes. Kumar et al., (2019) also isolated gallic acid and catechin from fab beans and reported that these phytochemicals showed better anti-diabetic results than acarbose [44].

MD simulation at 100 ns confirmed the virtual screening of natural bioactive compounds. When assessing the stability and dynamics of a protein-ligand complex, a popular computational method is molecular dynamics simulation. MD simulations of the initial structure of α-glucosidase-ligand (kaempferol and gallic acid) complexes were performed at 100 ns. A stable protein-ligand complex was formed, as evidenced by RMSD, RMSF, rGyr and solvent accessibility analysis. The overall secondary structure of glucosidase remained stable throughout the simulation, indicating that the protein-ligand complex remained stable.

In terms of inhibitory action against α-glucosidase, the docking score reveals that gallic acid is the most active molecule, followed by kaempferol. These potent phenolic compounds have important α-glucosidase inhibitory activity, shown in recent studies [45]. In addition to standard drugs, there are numerous synthetic inhibitors with higher α-glucosidase inhibitory activity [46]. Acarbose has also been reported as reference drug with IC_50_ [47,48]. The potential for anti-α-glucosidase seed extracts from *S. cumini* confirms a very active biological compound for a hyperglycemic drug that provides a safe and economical alternative. This study analyzed *S. cumini* seed extract through biochemical assay to identify their active compounds with anti-diabetic activities. Gallic acid and kaempferol showed IC_50_ at a concentration of 0.37 µM and 0.87 µM, respectively, as compared to acarbose (5.26 µM). Similar results were also found in *Hippophae rhamnoides* L. *Elaeagnaceae* leaves that stimulate the GLUT4 expression into adipocytes. It was concluded that sea buckthorn leaves play a key role in maintaining the blood glucose level in diabetic patients [49].

Compared to commercially available drugs, MD simulation reveals that bioactive compounds have potent α-glucosidase inhibitors. Due to the risk of adverse side effects and the higher cost of synthetic molecules, natural bioactive chemicals are preferred as potential drug candidates [50,51]. The majority of the inhibitors of α-glucosidase are effective at low concentration (IC_50_) than positive control and, therefore, these inhibitors may have excellent therapeutic values. Gallic acid has the most potent inhibitory activity, with the lowest IC_50_ value. New therapeutic techniques for the treatment of diabetic hyperglycemia, which are currently being researched, may benefit from these findings.

## 4. Materials and Methods

### 4.1. Collection and Selection of Plant Sample

*S. cumini* sample was purchased from the local market of Faisalabad, in the province of Punjab, Pakistan. The sample was thoroughly washed and dried in the fresh air. After 2–3 days, dry weight was calculated and this process was repeated until the plant sample had completely dried out. An electronic grinder was used to grind the sample to a fine powder.

### 4.2. Plant Extract Preparation

Approximately 10 g of *S. cumini* powder was dissolved in 30 mL of methanol and incubated at 37 °C in a shaking incubator overnight. After 24 h, the solution was vortexed for 10 min and the extract was filtered via Whatman filter paper into the Petri plate. The Petri plate was covered with porous aluminum foil to dry the filtrate and placed in a controlled environment. The dry extract from the Petri plates was scratched out after 24 h, measured and dissolved for further analysis into 500 μL of DMSO.

### 4.3. α-. Glucosidase Inhibitory Activity 

The α-glucosidase inhibitory activity is based on the p-Nitrophenol-α-D-glucopyranoside (PNPG) breakdown principle and used Taj, Ahmad [52] protocol with some modifications. About 12.5 µL of 200 µg/mL sample solution (dissolved in the DMSO) and 40 µL of 0.5 U/mL α-glucosidase from *Saccharomyces cerevisiae* (purchased from sigma Aldrich) were mixed in the 120 µL of 0.1 M Phosphate buffer (pH 7.4). After a 5-min incubation period, 40 µL of 5 mM PNPG solution was added to the reaction mixture, which was then incubated for 30 min at 37 °C. About 30 µL of 0.1 M Na_2_CO_3_ was added to terminate the reaction. At 405 nm, the microplate reader measured p-nitrophenol absorption. Acarbose (10 mM) was used as a positive control in this experiment. % Inhibition was computed with the following formula.


(1) $$\mathrm{Percentage}\;\mathrm{Inhibition}=\frac{\mathrm{Absorbance}\;\mathrm{of}\;\mathrm{control}\;–\;\mathrm{Absorbance}\;\mathrm{of}\;\mathrm{Sample}}{\mathrm{Absorbance}\;\mathrm{of}\;\mathrm{Control}}\times 100$$

Microdilution method was used to calculate the IC_50_ [27].

### 4.4. Molecular Docking

The 3D structure of the digestive enzyme α-glucosidase was downloaded from PDB (ID: 2QMJ). The retrieved 3D structure was optimized using Molecular Operating Environment (MOE) (MOE 2019.01) to remove solvent residues and ligand, 3D protonation and energy minimization [53]. Approximately 81 *S. cumini* phytochemicals were collected via PubChem and a literature search [54]. Phytochemicals were retrieved from PubChem in 3D structures and docked through the ready-to-dock library, which was formed by MOE. The active site of α-glucosidase was identified via MOE software. The active site was selected, which contains Asp (A542), Asp (A203), Asp (A327), His (A600) and Arg (A526) active amino acid residues. In the ready-to-dock library of plant chemicals, α-glucosidase residues interacted through the MOE. To achieve a minimum energy structure, the MOE program validates exact bond confirmation. Based on the RMSD value and S-score, the best and highest conformation compounds were determined after docking.

### 4.5. Drug-Likeness and ADMET Profiling 

A further evaluation of the most effective docking phytochemicals was carried out in accordance with Ro5 (Lipinski rule of five) [55]. According to the law, compounds must contain a maximum of five donors of hydrogen bonds and ten acceptors of hydrogen bonds, have less than 500 Daltons molecular weight and have an n-octanol/water partition coefficient (K_OW_) that must be less than 5. Mol. Inspiration server was utilized to measure its physicochemical properties and drug-like characteristics were determined through Swiss ADME [56]. ADMET properties are calculated to significantly indicate the drug-like behavior, toxicity and side effects in the human body. Pred-hERG (4.6) (http://predherg.labmol.com.br/) (accessed on 21 July 2022) was used to predict the cardiotoxicity against kaempferol and gallic acid. GUSAR (http://www.way2drug.com/gusar/acutoxpredict.htm) (accessed on 21 July 2022) software was used to evaluate the toxic level of drug and whether it was in an applicable domain or not.

### 4.6. Molecular Dynamics (MD) Simulation 

The influence of MD simulation in structural biology and the drug development process has increased day by day. MD simulation is a computer-based methodology to predict the movement of every atom of protein and the stability of protein to ligand complex under various circumstances. MD simulation was performed to confirm the docking results through Desmond v3.6 version. For this purpose, MD simulation was performed using a TIP3P water box (10 Å) with orthorhombic boundary conditions.

The protein ligand system was neutralized by adding sodium ions. Energy minimization of the protein ligand complexes were performed with the steepest descent (SD) method and LBFGS algorithms. The MD simulation was run with 100 ns via Desmond software for confirmation of the initial complexes of the protein-ligand, which were obtained from docking results. 

### 4.7. Statistical Analysis

All experiments were performed three times to obtain results representing the mean standard deviation (mean ± SD) using Microsoft Excel 2016. 

## 5. Conclusions

The current study reports the identification of two potent phytochemicals from *S. cumini* seeds that are effective as α-glucosidase inhibitory agents. The activity of gallic acid is greater than that of the other bioactive phytochemicals, kaempferol and acarbose. Here we can conclude that *S. cumini* seeds are a good source to control hyperglycemic conditions among diabetic patients and their top phytochemicals, gallic acid and kaempferol, can be used in combination with other synthetic anti-diabetic agents to stimulate drug activity with minimal dose and side effects.

## Figures and Tables

**Table 1:** Tabular depiction of IC_50_ of *Syzygium cumini* extract.

Sr. No.	Plant Extract	IC_50_
1	*Syzygium cumini*	8.07 (µg/mL)
2	Acarbose	5.26 (µM)

**Table 2:** Interaction detail of top two bioactive phytochemicals and control in the proposed site of α-glucosidase.

Sr. No.	PubChem ID	Chemical Name	Docking Score	RMSD Value	Receptor	Interaction
1	5,280,863	Kaempferol	−11.2	1.8	Asp542 Arg526 His600 Asp327	H-acceptor pi-H H-acceptor H-acceptor
2	370	Gallic acid	−10.3	1.0	Asp327 Asp547 His600	H-acceptor H-acceptor H-acceptor
3	41,774	Acarbose (Control)	−12.6	2.1	Asp542 Asp327 His600	H-acceptor H-acceptor H-acceptor

**Table 3:** Results of compounds examined for drug-likeness properties.

Compound	Molecular Weight (g/mol)	Number of HBA * (nON)	Number of HBD ** (nOHNH)	MlogP ***
Lipinski’s rule of five	<500	<10	<5	<5
Kaempferol	286.24	6	4	2.17
Gallic acid	170.12	5	4	0.59
Acarbose (Control)	645.61	19	14	−5.51

* HBA = hydrogen bond acceptor, ** HBD = hydrogen bond donor, *** MlogP = Moriguchi octanol-water partition coefficient.

**Table 4:** ADME Profiling of candidate phytocompounds.

Properties	Compounds	Acarbose	Gallic Acid	Kaempferol
Absorption	Water solubility	Soluble	Soluble	Soluble
	P-glycoprotein substrate	×	×	√
	Log Kp (skin permeation) cm/s	−16.29	−6.84	−8.73
Distribution	Blood-Brain Barrier	×	×	×
	Gastro-Intestinal Absorption	low	↑	↑
Metabolism CYP450	CYP4501 A2 Inhibitor	×	×	×
	CYP450 2C9 Inhibitor	×	×	×
	CYP450 2D6 Inhibitor	×	×	×
	CYP450 2C19 Inhibitor	×	×	×
	CYP450 3A4 Inhibitor	×	√	√
Excretion	Renal OCT2 substrate	No	No	No
Toxicity	Oral Toxicity (LD50) (mg/kg)	24,000	2851	475.5
	Oral Toxicity classification *	VI	V	IV

* class I: lethal if taken (LD50 ≤ 5); class II: Lethal if taken (5 less than LD50 ≤ 50); class III: lethal if taken (50 < LD50 ≤ 300); class IV: harmful if taken (300 < LD50 ≤ 2000); class V: minor side effects if taken (2000 < LD50 ≤ 5000); class VI: non-toxic (LD50 > 5000).

## Data Availability

Not applicable.
